# L'anneau de Kayser-Fleischer dans la maladie de Wilson

**DOI:** 10.11604/pamj.2018.30.137.15399

**Published:** 2018-06-18

**Authors:** Qariani Hajare, Khmamouche Mehdi

**Affiliations:** 1 Departement d'Ophtalmologie, Hôpital Militaire d'Instruction Mohammed V, Rabat, Maroc

**Keywords:** Maladie de wilson, anneau de Kayser-Fleischer, toxicose cuprique, Wilson's disease, Kayser-Fleischer ring, copper toxicosis

## Abstract

Wilson's disease is a copper toxicosis characterized by an overload in tissue copper (mainly hepatic, cerebral and pericorneal). It is caused by ATP7B mutations which incorporate copper into ceruloplasmin in the liver and pass out excess copper into the bile. This disease can be adequately treated if diagnosed early. The challenge is to diagnose it in the early stage of liver disease, before it becomes multisystemic. We report the case of a 19-year old female patient with Wilson's disease with multisytemic involvement: mucocutaneous pallor, intermittent hand tremor, that started when she was 16 years, hepatitis complicated by ascites, neurological disorder, such as postural tremor of the arms and the legs. Ophthalmologic examination using the slit-lamp showed Kayser-Fleischer ring in both eyes (A,B) which is a copper deposit in the peripheral corneal endothelium, more precisely on the border area between the cornea and the sclera (C). Laboratory tests showed low ceruloplasmin levels (0.03 g/L), cupremy 0.4 mg/l and urinary copper excretion 11.6 μmol. Brain MRI revealed generalized signal change, gliosis and atrophy of the thalamus and of the brainstem.

## Image en médecine

La maladie de Wilson est une toxicose cuprique caractérisée par une surcharge en cuivre tissulaire: essentiellement hépatique, cérébrale et péricornéenne .elle résulte de mutations sur l'ATP7B qui incorpore le cuivre à la céruléoplasmine dans le foie et libère le cuivre excédentaire dans la bile. Cette maladie peut être traitée de manière efficace si elle est diagnostiquée précocement. L'enjeu est d'en faire le diagnostic au stade initial de la maladie hépatique, avant qu'elle ne devienne multisystémique. Nous rapportons le cas d'une patiente de 19 ans qui présente une atteinte multisytémique dans le cadre de la maladie de wilson: une pâleur cutanéo-muqueuse, tremblement intermittent des mains depuis l'âge de 16 ans, une hépatite compliquée d'ascite, des troubles neurologiques à type d'un tremblement postural des bras et des jambes. L'examen ophtalmologique à la lampe à fente a objectivé au niveau des deux yeux l'anneau de Kayser-Fleischer (A,B) qui est un dépôt de cuivre à la périphérie de l'endothélium cornéen plus précisément sur la zone frontalière entre la cornée et la sclérotique(C). le bilan biologique trouve un taux de céruléoplasmine bas à 0,03 g/l; La cuprémie totale à 0,4 mg/l. l'excrétion urinaire de cuivre élevée à 11,6 μmol. L'imagerie par résonance magnétique cérébrale de la patiente a révélé un changement de signal généralisé, une gliose et une atrophie des thalamus et du tronc cérébral.

**Figure 1:**
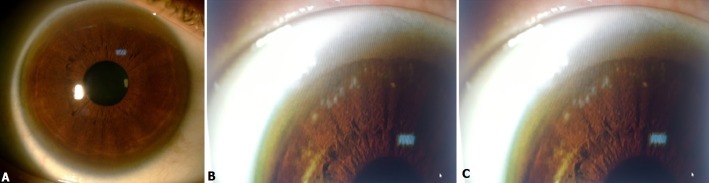
A) image de l'œil droit montrant l'anneau de Kayser Fleischer sur 360 de la périphérie cornéenne; B) image de l'œil gauche montrant l'anneau de Kayser Fleischer sur 360 de la périphérie cornéenne; C) fort grossissement de la périphérie cornéenne supéro-externe de l'œil droit

